# Morphological and Phylogenetic Analyses Reveal Four New Species of *Hydnellum* from China

**DOI:** 10.3390/jof12040267

**Published:** 2026-04-08

**Authors:** Yonglan Tuo, Yiming Li, Libo Wang, Hang Chu, Zhengxiang Qi, Jiajun Hu, Xiao Li, Bo Zhang, Yu Li

**Affiliations:** 1 Engineering Research Center of Edible and Medicinal Fungi Ministry of Education, Jilin Agricultural University, Changchun 130118, China; tuoyonglanfungi@163.com (Y.T.); shihanghangzhang@163.com (H.C.); qzx7007@126.com (Z.Q.); lxmogu@163.com (X.L.); 2 Hefei Mycological Valley Innovation Institute, Hefei 231100, China; 3 College of Life Sciences, Zhejiang Normal University, Jinhua 321000, China; hujifungi@163.com; 4 Sanjiang Laboratory, Jilin Agricultural University, Changchun 130118, China; 5 Industrial Development Institute for Plants, Animals and Fungi Integration of Biyang County, Biyang, Zhumadian 463799, China

**Keywords:** *Thelephorales*, *Bankeraceae*, 4 new taxa, stipitate hydnoid fungi

## Abstract

*Hydnellum* is an ectomycorrhizal fungus with important ecological and medicinal value. However, the species diversity of *Hydnellum* in China remains poorly understood. To deepen the understanding of the diversity of *Hydnellum* species in China, this study, based on a combination of morphological observations and molecular phylogenetic analysis of the internal transcribed spacer (ITS) and nuclear ribosomal large subunit (nrLSU) regions, identified and described four new species: *H. aureoluteum* sp. nov., *H. aureotomentosum* sp. nov., *H. fuscozonatum* sp. nov., and *H. pileospinosum* sp. nov. For each new species, we provided detailed morphological descriptions, hand-drawn illustrations, and comparisons with closely related taxa. In addition, this study systematically compiled key morphological characteristics and ecological distribution data for all known *Hydnellum* species in China and constructed a dichotomous identification key. This work provides an important basis for taxonomic research on the genus *Hydnellum* and enhances our understanding of its ecological distribution patterns in China.

## 1. Introduction

*Hydnellum*, an important genus within the *Bankeraceae*, was established by Karsten in 1879 [1]. All species in this genus are ectomycorrhizal fungi, forming symbiotic associations with diverse angiosperms and gymnosperms, particularly members of *Pinaceae* and *Fagaceae* [2,3]. They typically inhabit primary or minimally disturbed natural forests [3]. Through these mutualistic relationships, *Hydnellum* species enhance host plants’ absorption of water and mineral nutrients, while receiving photosynthetic products (carbohydrates) in return, thereby playing a crucial role in maintaining forest ecosystem health and facilitating vegetation restoration [4,5]. Furthermore, *Hydnellum* is endowed with significant medicinal potential due to its unique bioactive metabolites (e.g., polyphenols, terpenoids and p-terphenyls)—these components can exert health benefits through multiple mechanisms, including cholesterol-lowering, antioxidant, anti-inflammatory, antitumor, and anticoagulant effects [6,7,8]. For instance, extracts of *H. concrescens* have demonstrated favorable efficacy in adjuvant therapy for diabetes by inhibiting α-glucosidase activity [9], and their potential health value may be comparable to that of infusions from certain herbal plants [10]. However, affected by factors such as habitat loss, climate change, and soil acidification [11,12,13], the populations of *Hydnellum* species are declining sharply, raising global conservation concerns [14,15]. As a result, they have been included in the Red List of Endangered Species by countries including Norway, Poland, Germany, and the Netherlands [15,16,17]. Against this backdrop, a systematic analysis of the diversity characteristics and ecological distribution patterns of fungi in the genus *Hydnellum* is not only of key significance for their ecological conservation but also a necessary prerequisite for unlocking their potential in biotechnology, medical applications, and environmental science.

Morphologically, typical features of the genus *Hydnellum* are characterized by annual basidiomata with a zonate or an azonate pileal surface, spines—commonly white, orange, gray–blue, light brown, or dark brown—and usually stipitate basidiomata that are centrically or eccentrically attached [18]. Microscopically, a monomitic hyphal system is composed of generative hyphae with either simple septa or clamps. Basidiospores are generally subglobose to globose and bear tuberculate ornamentation [18,19]. Traditionally, both macroscopic traits (e.g., basidiocarp size, shape, color) and microscopic characteristics (e.g., spore dimensions, ornamentation) have been widely used for species identification in *Hydnellum* [19,20,21]. However, due to the high morphological similarity among many species, reliance on morphology alone is often insufficient for accurate species delimitation, even at the subgeneric level [22]. Regarding DNA-based molecular methods, ITS is still the most widely used region for identifying species within *Agaricomycetes*. In many groups of *Agaricales*, ITS may be sufficient to discriminate among species, whereas other (cryptic) taxa require secondary barcoding markers or multilocus analyses [23,24,25].

Recent studies integrating morphological and phylogenetic analyses (e.g., multi-locus sequencing) have significantly advanced taxonomic understanding of *Hydnellum* [26,27]. Typically, a new taxonomic group can be established when a group exhibits stable and distinctive morphological features (such as fruit body color, cap tomentum, annular zone characteristics; spore size, ornamentation, etc.), forms an independent clade in the phylogenetic tree (generally with Bayesian inference (BPP ≥ 0.95) and maximum likelihood analyses (MLBS ≥ 75%), and exhibits a clear genetic distance from closely related species [28,29,30]. In 2013, Baird et al. [18] assessed stipitate hydnoid fungi in the southern United States, identifying 41 taxa, of which 19 were *Hydnellum* species. In 2019, Larsson et al. [22] re-evaluated the generic boundary between *Hydnellum* and *Sarcodon* using ITS and nrLSU sequence data, thereby transferring 12 species from *Sarcodon* to *Hydnellum*. Chinese researchers have also contributed substantially: Mu et al. in 2021 [31] described 11 new *Hydnellum* species from China based on integrative taxonomy; Song et al. [32] reported five additional new species in 2022; Wang et al. [33] revised the *Hydnellum–Sarcodon* boundary and revealed eight phylogenetic species in *Hydnellum* in 2024; and Song et al. [34] in 2025 further explored molecular phylogeny and divergence times in *Thelephorales* using multi-locus markers (ITS + SSU + nrLSU + RPB2), describing 20 new species and proposing four new combinations. These efforts have markedly advanced *Hydnellum* taxonomy.

*Hydnellum* possesses a rich species diversity. According to Index Fungorum (Retrieved 27 February 2026), approximately 96 species have been described or transferred into this genus, which are predominantly distributed in North America and Europe [18,19,20,21,35,36,37]. Recent Chinese studies have enriched Asian diversity records, with 33 new *Hydnellum* species described from China—over half of which originate from Sichuan and Yunnan provinces [31,32,33,34,38,39]. This indicates that China, particularly its southwestern region, is a significant diversity center for *Hydnellum*. However, nationwide diversity patterns and potential distributions remain underexplored.

Between 2019 and 2024, we conducted extensive field surveys of *Hydnellum* resources across Northeast China, Central China, and Southwest China, collecting numerous specimens. By combining morphological observations and phylogenetic analyses based on ITS and nrLSU sequences, we identified and described four novel species of *Hydnellum*. The four *Hydnellum* species exhibit stable and unique morphological characteristics (e.g., spore size, pileus color, and habitat preference), which significantly distinguish them from known *Hydnellum* species. Phylogenetic analyses based on ITS and nrLSU sequences further revealed that each species forms a well-supported independent clade (BPP = 0.86–1.00, MLBS = 100%) and shows clear differentiation from closely related species. Additionally, we compiled the geographical distribution and key diagnostic morphological features of all confirmed *Hydnellum* species in China (Appendix A, Table A1), and compiled a dichotomous identification key for their discrimination. These data will facilitate the identification of *Hydnellum* and will contribute to a deeper understanding of the diversity patterns and ecological adaptations of Chinese *Hydnellum* species.

## 2. Materials and Methods

### 2.1. Morphological Studies

In this study, specimens were deposited in the Herbarium of Mycology, Jilin Agricultural University (HMJAU). The microscopic observation methods followed Hu et al. [40]. Sections were used for microscopic observation after cotton blue staining. Amyloid and dextrinoid reactions were detected using Melzer’s reagent [1.5 g KI (Sangan Biotech, Shanghai, China, A610443-0050), 0.5 g I_2_ (Sangan Biotech, Shanghai, China, A500538-0100), 20 g CCl_3_CH(OH)_2_ (Sangan Biotech, Shanghai, China, A500288-0250), dissolved in 20 mL distilled water]. Sections were mounted with 5% KOH (Sangan Biotech, Shanghai, China, A610441-0500), observed and measured under a Carl Zeiss Lab A1 microscope (Carl Zeiss AG, Jena, Germany) at a magnification of 1000×. Size measurements were performed using an eyepiece micrometer with an accuracy of 0.1 μm. For each specimen, the dimensions of at least 30 mature basidiospores, basidia, hyphae, and other microstructures were randomly measured. Spore size ranges were expressed in the format “(a) b–c (d)”, where “a” and “d” represent the minimum and maximum measured values, respectively, and 95% of the spores fell within the interval “b–c”. Abbreviations used in this paper are defined as follows: av. L = average spore length; av. W = average spore width; Q = length-to-width ratio, av. Q = average length-to-width ratio; MR = Melzer’s reagent (MR+ = amyloid reaction; MR− = nonamyloid); and LCB = Lactophenol Cotton Blue (LCB+ = cyanophilous; LCB− = acyanophilous) (Yishijiu BIOISCO, Lianyungang, Jiangsu, China, SM020-50). Spore ornamentation was observed using a field emission scanning electron microscope (FESEM; JSM-IT800, JEOL, Tokyo, Japan) with an accelerating voltage of 5 kV. Colors were referenced to the Methuen Handbook [41]. Additionally, this study designed a dichotomous identification keybased on the main morphological characteristics of known *Hydnellum* species in China, including cap color and size, tooth color and length, spore size, and habitat preference, among others. This key aims to assist users in quickly and accurately identifying these species, thereby providing a reliable basis for subsequent ecological surveys, resource utilization, or conservation research (Appendix A, Table A1).

### 2.2. DNA Extraction, PCR, and Sequencing

Genomic DNA was extracted from dried specimens using the NuClean Plant Genomic DNA Kit (Cat. No. CW0531M, Cowin Biotech Co., Ltd., Taizhou, Jiangsu, China). The internal transcribed spacer (ITS) was amplified using the primer pair ITS1F/ITS4, and the large subunit of the nuclear ribosomal RNA gene (nrLSU) was amplified using the primer pair LR0R/LR5 [31]. The PCR reaction system was 15 μL, consisting of: 1.5 μL template DNA, 1 μL forward primer (10 μM), 1 μL reverse primer (10 μM), 4 μL ddH_2_O, and 7.5 μL SanTaq^®^ PCR Master Mix (Shanghai Sangon Biotech Co., Ltd., Shanghai, China; Cat. No. B532081-0020). For the ITS region, the PCR amplification program was as follows: initial denaturation at 95 °C for 4 min; followed by 35 cycles of denaturation at 95 °C for 45 s, annealing at 56 °C for 45 s, and extension at 72 °C for 1.5 min; and a final extension at 72 °C for 10 min. For the nrLSU region, the PCR amplification program was: initial denaturation at 95 °C for 3 min; followed by 35 cycles, each comprising denaturation at 95 °C for 45 s, annealing at 58 °C for 60 s, and extension at 72 °C for 1.5 min; and a final extension at 72 °C for 7 min. The PCR products were sent to Jilin Kumei Biotechnology Co., Ltd. (Changchun, China) for bidirectional Sanger sequencing. The sequencing results were assembled using SeqMan software (DNASTAR Inc., v7.1), and the final sequences were submitted to the GenBank database (https://www.ncbi.nlm.nih.gov/genbank/, accessed on 13 January 2026). The aforementioned primers, ITS1F/ITS4 and LR0R/LR5, were both synthesized by Jilin Kumei Biotechnology Co., Ltd.

### 2.3. Phylogenetic Inference

Sequences for phylogenetic analysis were obtained from three sources: (1) existing sequences in relevant taxonomic studies [31,32,33,42], (2) sequences downloaded from GenBank, and (3) newly generated sequences in this study (Table 1). *Sarcodon muscicola*, *S. leucopus*, *S. scabripes*, and *S. squamosus* were used as outgroups [31]. Phylogenetic analyses were performed based on a concatenated dataset of the internal transcribed spacer (ITS) and nuclear large subunit ribosomal DNA (nrLSU) gene regions. The analytical workflow was implemented in PhyloSuite v1.2.3 [43] as follows: (1) sequences of the ITS and nrLSU regions were aligned separately using the MAFFT program. After manual adjustment of the alignments, the resulting aligned sequences were concatenated; (2) based on the Bayesian Information Criterion (BIC), the best-fit evolutionary models for Bayesian inference (BI) and maximum likelihood (ML) analyses were selected using the ModelFinder program integrated within PhyloSuite; (3) Bayesian inference (BI) was performed using MrBayes, and maximum likelihood (ML) analysis was conducted using IQ-TREE; and (4) the resulting phylogenetic tree was visualized using the online tool iTOL (v7.2.1), and subsequently edited and formatted with Adobe Illustrator 2020 (Adobe, San Jose, CA, USA).

## 3. Results

### 3.1. Phylogenetic Analyses

**Figure 1:**
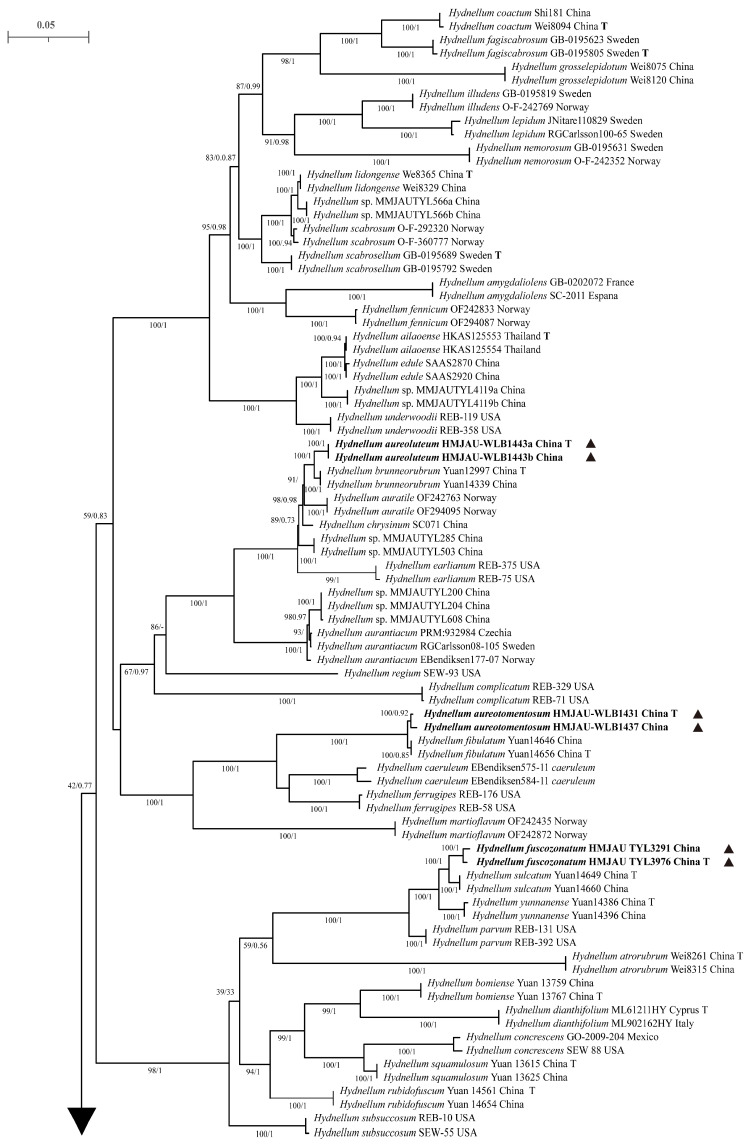
Phylogenetic tree of *Hydnellum* inferred from Bayesian and maximum likelihood analyses based on the combined nrLSU + ITS dataset. Node support is shown as the maximum likelihood bootstrap support (MLBS, left) ≥ 70% and the Bayesian posterior probability (BPP, right) ≥ 0.95. Holotype specimens are marked with T. New taxa are marked with a solid triangular symbol (▲).

**Figure 2:**
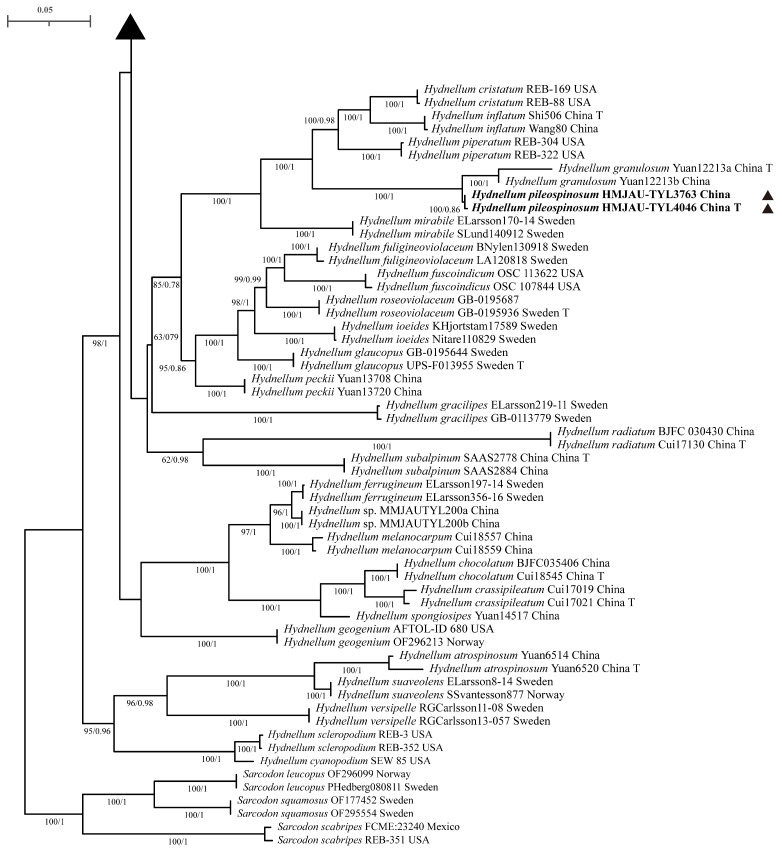
Phylogenetic tree of *Hydnellum* inferred from Bayesian and maximum likelihood analyses based on the combined nrLSU + ITS dataset. Node support is shown as the maximum likelihood bootstrap support (MLBS, left) ≥ 70% and the Bayesian posterior probability (BPP, right) ≥ 0.95. Holotype specimens are marked with T. New taxa are marked with a solid triangular symbol (▲).

In this study, phylogenetic trees were constructed using 249 sequences from two gene markers (nrLSU and ITS). Among these, 38 sequences were newly obtained through sequencing, including 19 nrLSU sequences and 19 ITS sequences. The combined nrLSU + ITS dataset comprised 72 taxa and 5273 sites, including 1713 (32.49%) parsimony-informative sites, 234 singleton sites (4.44%), and 3326 (63.08%) constant sites. For Bayesian inference (BI), the best-fit partitioned model for nrLSU + ITS was GTR + I + G4 + F, with 5 million generations, an average ESS of 1405.79, and a potential scale reduction factor of 1.000. For maximum likelihood (ML) analysis, the best-fit partitioned model was also GTR + F + I + R4 (for nrLSU + ITS), with 1000 bootstrap replicates performed. As the topological structures inferred by ML and BI analyses were similar, only the ML tree is presented here (Figure 1 and Figure 2).

The results showed that four novel species—*H. aureoluteum* (MLBS = 100%, BPP = 1), *H. aureotomentosum* (MLBS = 100%, BPP = 0.92), *H. fuscozonatum* (MLBS = 100%, BPP = 1), and *H. pileospinosum* (MLBS = 100%, BPP = 0.86)—were divided into four well-supported species-level clades. Specifically, *H. aureotomentosum* formed a sister-group relationship with *H. fibulatum* (MLBS = 100%, BPP = 1); *H. aureoluteum* formed a sister-group relationship with *H. brunneorubrum* (MLBS = 100%, BPP = 1); and *H. fuscozonatum* formed a sister-group relationship with *H. sulcatum* (MLBS = 100%, BPP = 1). The other five new taxa (not formally described and to be published later) also form five independent species-level clades, and all have high support values: *Hydnellum* sp. 1 (MLBS = 100%, BPP = 1); *Hydnellum* sp. 2 (MLBS = 100%, BPP = 1); *Hydnellum* sp. 3 (MLBS = 100%, BPP = 1); *Hydnellum* sp. 4 (MLBS = 100%, BPP = 1); *Hydnellum* sp. 5 (MLBS = 100%, BPP = 1).

### 3.2. Taxonomy

**Figure 3:**
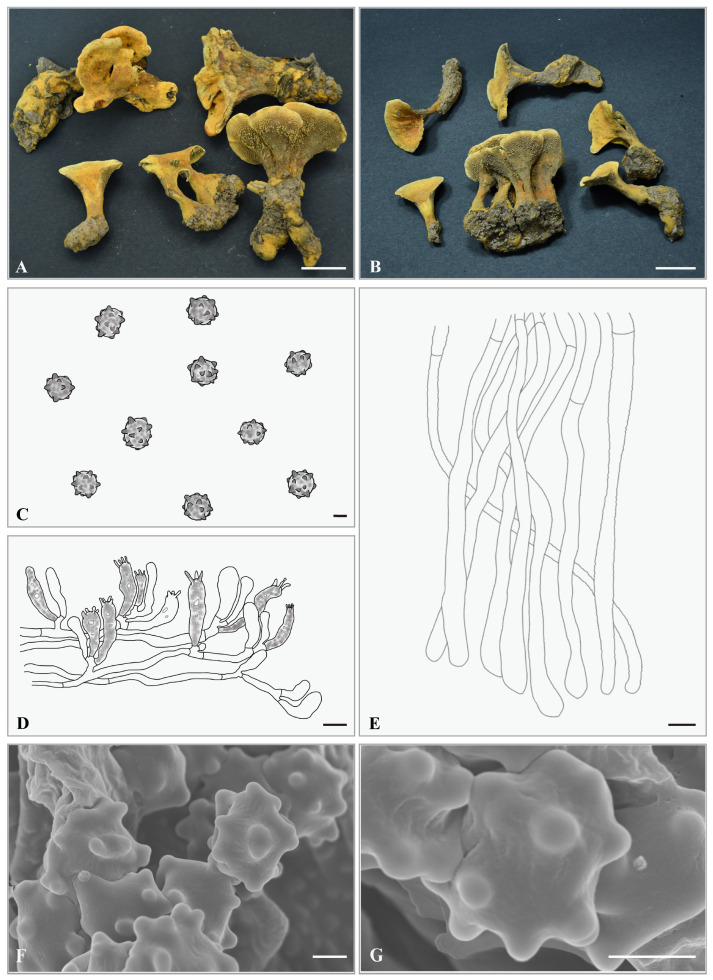
*Hydnellum aureoluteum*. (**A**,**B**) Basidiocarps ((**A**) = HMJAU-WLB1443a, (**B**) = HMJAU-WLB1443b); (**C**–**E**) microscopic structures (drawn from HMJAU-WLB1443a); (**C**) basidiospores; (**D**) hymenium and subhymenium; (**E**) pileipellis; (**F**,**G**) SEM of basidiospores (SEM images from HMJAU-WLB1443a). Scale bar: (**A**,**B**) = 2 cm; (**C**,**F**,**G**) = 2 μm; (**D**,**E**) = 10 μm.

***Hydnellum aureoluteum*** Yonglan Tuo, Bo Zhang & Yu Li sp. nov. Figure 3A–G.

**Fungal Name**: FN 573273.

**Etymology.** The specific epithet “*aureoluteum*” refers to the golden-yellow coloration of the entire basidiocarps.

**Holotype. CHINA.** Sichuan Province, Tongjiang City, 32°7′50″ N, 107°7′4″ E, elevation: ca. 662 m, on soil in a *Q. acutissima* forest, 29 July 2024, Libo Wang (HMJAU-WLB1443a). GenBank accession numbers: ITS: PX856753, nrLSU: PX855234.

**Diagnosis.** *H. aureoluteum* differs from other *Hydnellum* species in that its entire basidiocarp exhibits a golden-yellow coloration from youth to maturity.

**Description.** Basidiocarps small, gregarious, 25.5–48.5 mm in height. Pileus 16.5–30.3 mm wide, irregularly circular to flabelliform, depressed centrally, golden yellow (5B7); surface pubescent to floccose when fresh; margin incurved and covered with orange gray (5B2) tomentum. Pileus context 2.0–4.5 mm thick, orange gray (5B2). Spines not decurrent, grayish orange (5B5) to light brown (6D5), slightly sparse, 1–2 spines/mm^2^, 0.5–1.5 mm long, 0.1–0.25 mm in diameter. Stipe 14.5–40.5 × 4.0–10.5 mm, golden yellow (5B7) to light brown (6D5), covered with golden-yellow (5B7) tomentum, usually eccentric, cylindrical and somewhat inflated at the base, solid. Odor mild or fruity.

Basidiospores 4.0–5.0 × 4.0–4.5 (5.0) μm, av. L = 4.57 μm, av. W = 4.03 μm, Q = 1.00–1.25, av. Q = 1.13, globose to subglobose, light brown, thin-walled, tuberculate, MR−, LCB+; tuberculi usually isolated, 0.6–1.1 μm long, 0.6–1.0 μm in diameter. Basidia 24.5–30 × 6.0–7.0 μm, clavate to suburniform, some with granular contents; sterigmata 2.5–3.0 × 0.5–1.0 µm. Basidioles 15.0–25.0 × 5.0–6.0 μm, smaller than basidia, some with granular contents. Subhymenium trama filamentous, hyphae 3.0–3.5 μm wide, septate, thin-walled, hyaline in 5% KOH. Hyphae of pileus context 4.0–5.0 μm, thin-walled. Pileipellis composed of cylindrical hyphae, subparallel, rarely branched, terminal elements cylindrical at apex, cells 40.5–120.5 × 5.0–6.0 μm. Hyphae from the inner layer of stipe, 4.5–5.5 μm wide, subparallel, occasionally branched. Hyphae from the surface layer of stipe, 4.0 μm wide, interwoven, occasionally branched. Clamp connections not observed.

**Habitat and distribution.** This species was observed growing gregariously in a *Q. acutissima* forest within the Tongjiang City, Sichuan Province, China (ca. 600–700 m, Subtropical Monsoon Climate), an area with human disturbance. Two specimens were collected at distances of 54.5 cm (DBH = 20.2 cm) and 42.5 cm (DBH = 18.5 cm) from a *Q. acutissima* tree.

**Additional specimens examined. CHINA.** Sichuan Province, Tongjiang City, 32°7′48″ N, 107°7′6″ E, elevation: ca. 652 m, on soil in a *Q. acutissima* forest, 29 July 2024, Libo Wang (HMJAU-WLB1443b). GenBank accession numbers: ITS: PX856754, nrLSU: PX855235.

**Notes.** *H. aureoluteum* and *H. brunneorubrum* are very similar in pileus size (16.5–30.3 mm vs. 40 mm), basidiospores size (av. L × av. W = 4.57 μm × 4.03 μm vs. 4.9 μm × 3.9 μm), and spine color (grayish orange (5B5) to light brown (6D5)). However, *H. aureoluteum* is characterized by an entirely golden-yellow basidiocarp, whereas the pileal surface of *H. brunneorubrum* is brownish orange (6C8) to brownish red (10D8). Furthermore, consistent differences are observed between the ITS and nrLSU sequences of the two species.

**Figure 4:**
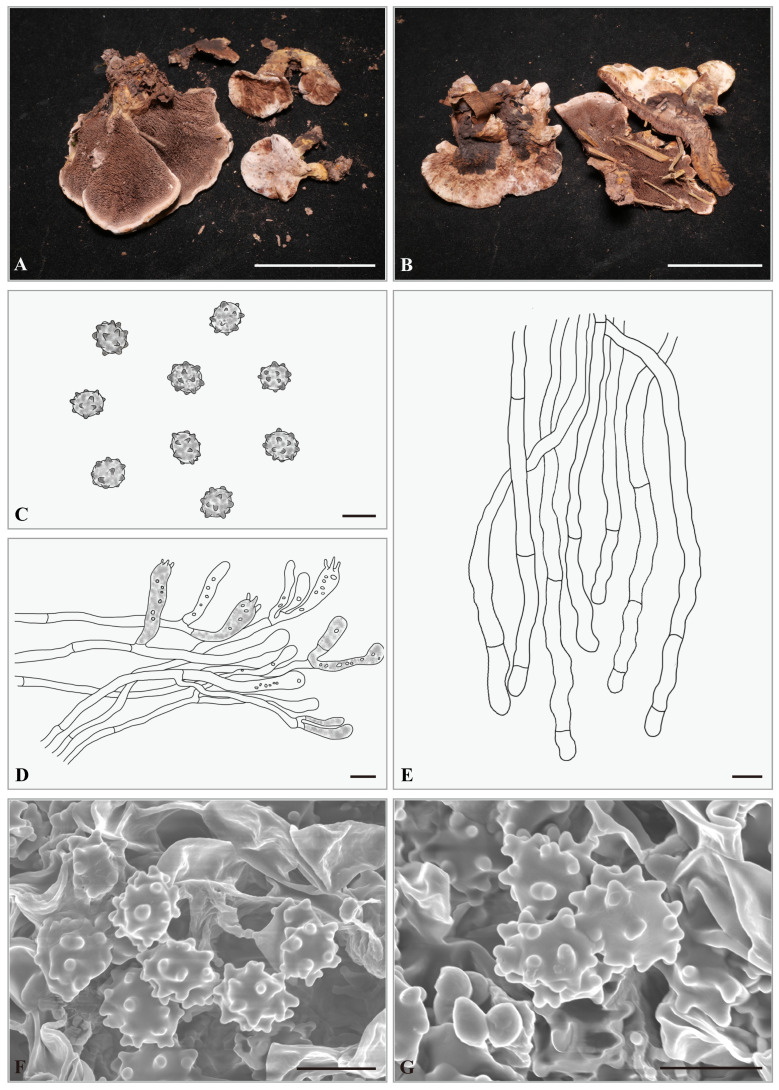
*Hydnellum aureotomentosum*. (**A**,**B**) Basidiocarps ((**A**) = HMJAU-WLB1431, (**B**) = HMJAU-WLB1437); (**C**–**E**) microscopic structures (drawn from HMJAU-WLB1431); (**C**) Basidiospores; (**D**) hymenium and subhymenium; (**E**) pileipellis; (**F**,**G**) SEM of basidiospores (SEM images from HMJAU-WLB1431). Scale bar: (**A**,**B**) = 2 cm; (**C**,**F**–**G**) = 2 μm; (**D**,**E**) = 10 μm.

***Hydnellum aureotomentosum*** Yonglan Tuo, Bo Zhang & Yu Li sp. nov. Figure 4A–G.

**Fungal Name:** FN 573272.

**Etymology.** The specific epithet “*aureotomentosum*” refers to the golden-yellow tomentum that densely covers the stipe surface of this species.

**Holotype. CHINA.** Sichuan Province, Tongjiang City, 32°8′5″ N, 107°6′44″ E, elevation: ca. 732 m; on soil in a *Q. acutissima* Carruth. forest, 29 July 2024, Libo Wang (HMJAU-WLB1431). GenBank accession numbers: ITS: PX856755, nrLSU: PX855236.

**Diagnosis.** *H. aureotomentosum* differs from other *Hydnellum* species in having the stipe surface densely covered with golden-yellow tomentum from the young to the mature stage.

**Description.** Basidiocarps, small to medium, solitary to gregarious, 12.5–35.5 mm in height, leathery when fresh. Pileus 19.5–75.5 mm broad, orange gray (6B2) to brown (6E7), covered with orange gray (6B2) tomentum, which becomes brown (6E7) upon contact. Pileus context 2.4–6.5 mm thick, woody, light brown (6D5). Spines not decurrent, conical, brown (6E7), slightly crowded, 1–2 spines/mm^2^, 2.0–4.5 mm long, 0.10–0.25 mm in diameter. Stipe 12.0–35.5 × 6.0–10.5 mm, covered with golden-yellow (5B7) tomentum, leathery when fresh, central, cylindrical, solid. Odor mild.

Basidiospores (5.0) 5.5–6.0 × 5.0–5.5 (6.0) μm, av. L = 5.91 μm, av. W = 5.04 μm, Q = 1.0–1.2, av. Q = 1.17, globose to subglobose, light brown (6D4), thin-walled, tuberculate, MR−, LCB−; tuberculi usually isolated or grouped in pairs, 0.4–0.9 μm long and 0.5–1.1 μm wide. Basidia 32.0–40.0 × 7.0–8.0 μm, clavate; sterigmata 3.0–4.0 µm long. Basidioles 15.0–25.0 × 5.0–7.0 μm, smaller than basidia. Subhymenium trama filamentous, hyphae 3.0–4.0 μm wide, thin-walled, subparallel, branched. Hyphae of pileus context 4.0–5.0 μm, thin-walled. Pileipellis composed of cylindrical hyphae, subparallel, rarely branched, terminal elements cylindrical at apex, cells 30.0–105.0 × 6.0–7.0 μm. Hyphae from the inner layer of stipe, 4.0–5.0 μm wide, subparallel. Hyphae from the surface layer of stipe, 4.0–5.0 μm wide, subparallel, occasionally branched.

**Habitat and distribution.** The species occurs solitary to gregarious in *Q. acutissima* forest (ca. 700–800 m). The forest is subject to human disturbance (grazing and thinning). Two specimens were collected at distances of 45.6 cm (DBH = 12.8 cm) and 54.5 cm (DBH = 18.6 cm) from *Q. acutissima* trees.

**Additional specimens examined. CHINA.** Sichuan Province, Tongjiang City, 32°8′5″ N, 107°6′8″ E, elevation: ca. 763 m; on soil in a *Q. acutissima* forest, 29 July 2024, Libo Wang (HMJAU-WLB1437). GenBank accession numbers: ITS: PX856756, nrLSU: PX855237.

**Notes.** Morphologically, *H. aureotomentosum* resembles *H. fibulatum* in having small to medium basidiocarps with spines that are brown (6E7) and not decurrent. However, *H. aureotomentosum* differs in having an orange-gray (6B2) pileus (whereas *H. fibulatum* has a pileus ranging from light brown (7D7) to dark brown (8F4)). Furthermore, *H. aureotomentosum* has a larger basidiospore size (av. L × av. W = 5.91 × 5.04 μm vs. 5.2 × 4.3 μm). Phylogenetic analysis indicates that *H. aureotomentosum* forms a distinct lineage sister to *H. fibulatum*. Based on this combined morphological and phylogenetic evidence, *H. aureotomentosum* is readily distinguishable from other *Hydnellum* species.

**Figure 5:**
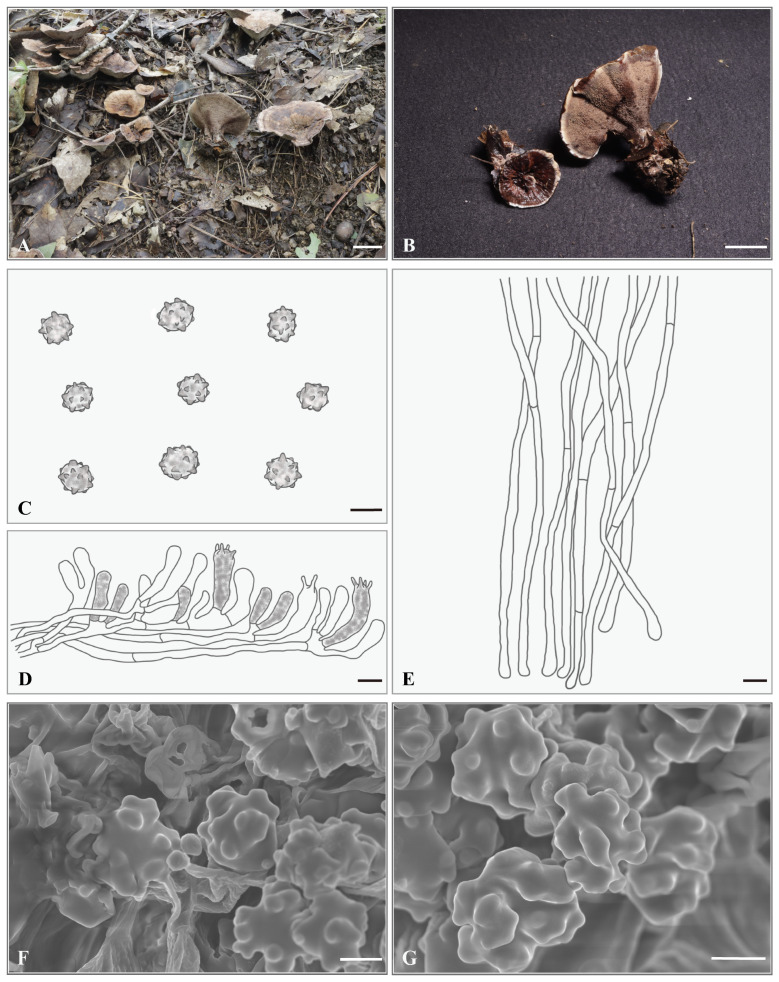
*Hydnellum fuscozonatum*. (**A**,**B**) Basidiocarps ((**A**) = HMJAU-TYL3976, (**B**) = HMJAU-TYL3291); (**C**–**E**) microscopic structures (drawn from HMJAU-TYL3976); (**C**) Basidiospores; (**D**) hymenium and subhymenium; (**E**) pileipellis; (**F**,**G**) SEM of basidiospores (SEM images from HMJAU-TYL3976). Scale bar: (**A**,**B**) = 2 cm; (**C**) = 5 μm; (**D**,**E**) = 10 μm; (**F**,**G**) = 2 μm.

***Hydnellum fuscozonatum*** Yonglan Tuo, Bo Zhang & Yu Li sp. nov. Figure 5A–G.

**Fungal Name:** FN 573271.

**Etymology.** The specific epithet “*fuscozonatum*” refers to the characteristic brown zonate bands on the pileus of this species.

**Holotype. CHINA.** Anhui Province, Lu’an City, Tianma National Nature Reserve, 31°9′37″ N, 115°50′56″ E, elevation: ca. 715 m; on soil in a *Q. glauca* Thunb. forest, 8 October 2023, Yonglan Tuo (HMJAU-TYL3976). GenBank accession numbers: ITS: PX856758, nrLSU: PX855239.

**Diagnosis.** *H. fuscozonatum* differs from other *Hydnellum* species by a pileus surface with brown zonation, a margin covered with white tomentum, and storeyed basidiocarps.

**Description.** Basidiocarps, small to medium, solitary to gregarious, storeyed, 15.3–64.2 mm in height, leathery when fresh. Pileus 12.5–65.4 mm broad, gray (7B1) to brownish gray (7F3), circular to infundibuliform, brown-zonate, and covered with white (7A1) tomentum. Pileus context 2.5–3.5 mm thick, woody, grayish (6D3). Spines decurrent, conical, white (7A1) to brown (6E5), becoming dark brown (6F5) when touched, slightly crowded, 2–3 spines/mm^2^, 0.5–2.5 mm long, 0.25–0.5 mm in diameter. Stipe 12.0–36.5 × 12.0–16.4 mm, covered with gray (7B1) to grayish brown (6D3) tomentum, which becomes water-soaked upon contact and turns brown, leathery when fresh, central, cylindrical, with a distinctly swollen base, solid. Odor mild.

Basidiospores 5.5–6.0 × 5.0–5.5 μm, av. L = 5.94 μm, av. W = 5.02 μm, Q = 1.09–1.20, av. Q = 1.18, subglobose, brown (6E5), thin-walled, tuberculate, MR−, LCB−; tuberculi usually isolated or grouped in 2–3s, 0.25–0.7 μm long. Basidia 25.0–35.0 × 7.0–8.0 μm, clavate to cylindrical; sterigmata 2–4, 2.5–3.0 × 0.25–0.5 µm. Basidioles 15.0–28.5 × 5.0–6.0 μm, smaller than basidia. Subhymenium trama filamentous, hyphae 3.0 μm wide, septate, thin-walled, light brown (6D4) in 3% KOH. Hyphae of pileus context 4.0–5.0 μm, thin-walled. Pileipellis composed of cylindrical hyphae, subparallel, rarely branched, terminal elements cylindrical at apex, cells 69.0–115.5 × 4.5–5.0 μm. Hyphae from the inner layer of stipe, 4.0–5.0 μm wide, subparallel, occasionally branched. Hyphae from the surface layer of stipe, 4.0 μm wide, slightly interwoven, occasionally branched.

**Habitat and distribution.** The species occurs solitary to gregarious in *Q. glauca* forest within the buffer zone of Tianma National Nature Reserve, China (ca. 600–800 m, subtropical monsoon climate). The forest is subject to human disturbance (thinning, and proximity to villages and towns at approximately 0.2–4.5 km). Specimens were collected at distances of 105.4 cm (DBH = 12.5 cm) and 125.5 cm (DBH = 25.5 cm) from a *Q. glauca* tree.

**Additional specimens examined. CHINA.** Anhui Province, Lu’an City, Tianma National Nature Reserve, 31°9′35″ N, 115°50′58″ E, elevation: ca. 662 m; on soil in a *Q. glauca* forest, 20 September 2023, Yonglan Tuo (HMJAU-TYL3291). GenBank accession numbers: ITS: PX856757, nrLSU: PX855238.

**Notes.** Morphologically, *H. fuscozonatum* is similar to *H. sulcatum*, both possessing solitary to gregarious basidiocarps and having a brown zonate pileus. However, *H. fuscozonatum* differs in having larger basidiospores (av. L × av. W = 5.94 × 5.02 μm vs. 4.8 × 4.3 μm); its pileus margin surface and inner edge are covered with brownish orange (5C3) tomentum, which becomes water-soaked upon contact and subsequently turns dark brown (6F5).

In the phylogenetic tree, the sequence of *H. fuscozonatum* forms a distinct lineage, which, together with *H. sulcatum*, *H. yunnanense*, *H. parvum*, and *H. subsuccosum*, forms a highly supported major subclade.

**Figure 6:**
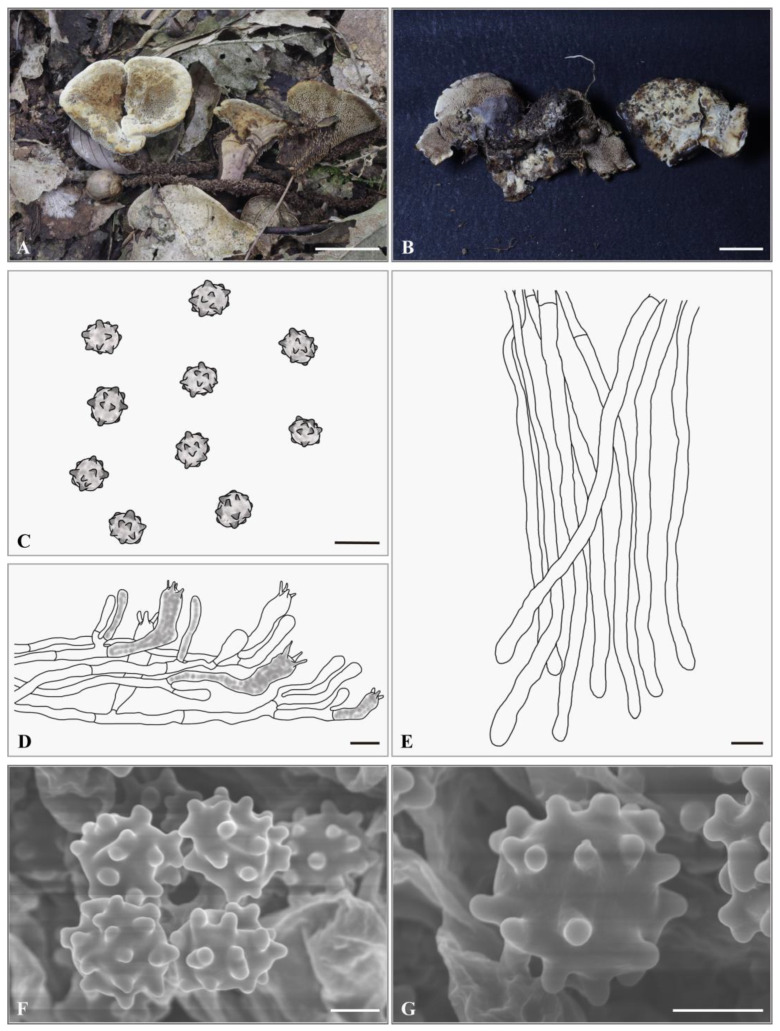
*Hydnellum pileospinosum*. (**A**,**B**) Basidiocarps ((**A**) = HMJAU-TYL3763, (**B**) = HMJAU-TYL4046); (**C**–**E**) microscopic structures (drawn from HMJAU-TYL4046); (**C**) Basidiospores; (**D**) hymenium and subhymenium; (**E**) pileipellis; (**F**,**G**) SEM of basidiospores (SEM images from HMJAU-TYL4046). Scale bar: (**A**,**B**) = 2 cm; (**C**) = 5 μm; (**D**,**E**) = 10 μm; (**F**,**G**) = 2 μm.

***Hydnellum pileospinosum*** Yonglan Tuo, Bo Zhang & Yu Li sp. nov. Figure 6A–G.

**Fungal Name:** FN 573270.

**Etymology.** The specific epithet “*pileospinosum*” refers to the presence of spines on the pileus.

**Holotype. CHINA.** Anhui Province, Lu’an City, Tianma National Nature Reserve, 31°10′5″ N, 115°50′58″ E, elevation: ca. 882 m; on soil in a *Q*. *glauca* Thunb. forest, 27 September 2023, Yonglan Tuo (HMJAU-TYL3763). GenBank accession numbers: ITS: PX856759, nrLSU: PX855240.

**Diagnosis.** *H. pileospinosum* differs from other *Hydnellum* species in having smaller basidiospores (4.0–5.0 × 4.0–4.5 μm) and spines attached to the pileus.

**Description.** Basidiocarps, solitary, small to medium, 25.3–30.6 mm in height, leathery when fresh. Pileus 26.2–60.3 mm broad, surface pubescent to floccose when fresh, grayish yellow (4B3) to yellowish brown (5E5), circular to sectoral, center depressed; pileus margin with white (4A1) to grayish yellow (4B3) tomentum, center covered with yellowish brown (5E5) tomentum, partially with short spines attached. Pileus context 5.0–10.0 mm thick, woody, yellowish brown (5E5). Spines decurrent, conical, white (6A1) to brown (6E4), spine tips white (4A1), slightly crowded, 2–3 spines/mm^2^, 1.0–6.7 mm long, 0.25–0.5 mm in diameter. Stipe 15.0–20.5 × 5.0–12.5 mm, covered by short spines, leathery when fresh, central, cylindrical to attenuate below, solid. Odor mild.

Basidiospores 4.0–5.0 × 4.0–4.5 μm, av. L = 4.41 μm, av. W = 4.02 μm, Q = 1.00–1.13 (1.25), av. Q = 1.09, globose to subglobose, brown (6E5), thin-walled, tuberculate, MR+, LCB+; tuberculi usually isolated, 0.25–0.5 μm long. Basidia 30.0–36.5 × 6.5–7.5 μm, clavate to cylindrical; sterigmata 2–4, 3.5–5.0 × 0.25–0.5 µm. Basidioles 20.0–28.5 × 6.5–7.0 μm, smaller than basidia. Subhymenium trama filamentous, hyphae 3.5–4.0 μm wide, septate, thin-walled, hyaline in 3% KOH. Hyphae of pileus context 6.0–6.5 μm, thin-walled. Pileipellis composed of cylindrical hyphae, slightly interwoven, rarely branched, terminal elements cylindrical at apex, cells 60.5–140.5 × 5.0–5.5 μm. Hyphae from the inner layer of stipe, 5.5–6.0 μm wide, subparallel, occasionally branched. Hyphae from the surface layer of stipe, 4.5–5.0 μm wide, slightly interwoven, occasionally branched.

**Habitat and distribution.** The species occurs solitary to gregarious in a *Q. glauca* forest within the buffer zone of Tianma National Nature Reserve, China (ca. 700–900 m, subtropical monsoon climate). The forest is subject to human disturbance (grazing, thinning, and proximity to villages and towns at approximately 0.2–3.5 km). Two specimens were collected from the *Q. glauca* trees at distances of 78.2 cm (DBH = 30.2 cm) and 32.5 cm (DBH = 27.6 cm), respectively.

**Additional specimens examined. CHINA.** Anhui Province, Lu’an City, Tianma National Nature Reserve, 31°9′58″ N, 115°50′58″ E, elevation: ca. 744 m; on soil in a *Q. glauca* forest, 6 October 2023, Yonglan Tuo (HMJAU-TYL4046). GenBank accession numbers: ITS: PX856760, nrLSU: PX855241.

**Notes.** Morphologically, *H. pileospinosum* resembles *H. granulosum* in having solitary to gregarious basidiocarps with a subinfundibuliform to circular pileus, a brown cylindrical stipe, and basidiospores of similar shape and size. However, *H. pileospinosum* differs in having a larger pileus (up to 60.3 mm in diameter, compared to up to 50 mm in *H. granulosum*), a pileus covered with tomentum ranging from white (4A1) to grayish-yellow (4B3), short and partially attached spines, and longer spines (6.7 mm, compared to 2.0 mm in *H. granulosum*). Furthermore, the habitats of the two species differ significantly: *H*. *granulosum* inhabits *Acer* and *Cryptomeria* mixed forests, whereas *H. pileospinosum* occurs in single-species *Q. glauca* forests.

In the phylogenetic tree, the sequences of *H. pileospinosum* cluster together, forming a distinct lineage, and form a sister clade to *H. granulosum*.

## 4. Discussion

Based on the integration of morphological observations and molecular phylogenetic analyses, this study confirmed and described four new species of *Hydnellum* (*H. aureoluteum*, *H. aureotomentosum*, *H. fuscozonatum*, and *H. pileospinosum*), expanding the number of currently known *Hydnellum* species in China to 48 and further enriching the understanding of *Hydnellum* diversity. Additionally, we summarized the key morphological and ecological characteristics of *Hydnellum* species recorded from China and added an identification key (Table 2) to facilitate their accurate identification and deepen the understanding of their ecological habits.

Typically, combinations of morphological traits are effective for distinguishing closely related species [19,21,45,46,47]. In this study, four newly described taxa can also be distinguished from closely related species through a combination of macroscopic features (e.g., pileus ornamentation, size, and color) and microscopic features (e.g., spore and basidia size). For example, *H. aureotomentosum* is characterized by an orange-gray pileus, distinguishing it from its sister taxon *H. fibulatum* [31], which exhibits a pileus coloration ranging from light brown to dark brown; the spores of *H. aureotomentosum* are also relatively larger (av. L × av. W = 5.91 × 5.04 vs. 5.2 × 4.3 μm). However, overlap of morphological features exists among some species of *Hydnellum*. For instance, *H. chocolatum* [32] and *H. crassipileatum* [32] exhibit extremely similar characteristics in terms of basidiocarp size, shape, and color; and spore size (5.0–6.0 × 4.0–5.0 μm vs. 4.0–6.0 × 4.0–5.5 μm), season (September), and habitat (mixed forests). Consequently, accurate identification of many *Hydnellum* species based solely on morphological characteristics is difficult or even impossible, which has constrained taxonomic research within this genus.

In contrast, an integrated approach combining macroscopic and microscopic observations and molecular sequence analysis has demonstrated high effectiveness in distinguishing species within the genus *Hydnellum* [22,33,34,48]. During 2021–2025 alone, 33 new *Hydnellum* species were described in China [31,32,33,34,38,39]. In this study, the phylogenetic tree constructed based on ITS + nrLSU sequences reveals that all four new species form independent evolutionary clades with extremely high support values (BS = 100%, BPP = 0.93–1.00), and they are also significantly distinguishable from other species within the genus morphologically. This indicates that through an integrative taxonomic approach combining morphological, molecular, and ecological data, more potential species of *Hydnellum* can be discovered [34,42,49]. Such an approach will also facilitate future phylogenetic studies of *Hydnellum* to establish a more robust classification system.

The distribution of *Hydnellum* species appears to be relatively limited within their host plant communities [2,15,42,50]. Specimens in this study were collected from *Quercus* forests composed of a single tree species, with collection sites located 30–130 cm away from oak trees. By comparing the recorded collection localities of *Hydnellum* species in China (Appendix A, Table A1), we found that forests of *Pinaceae* and *Fagaceae* serve as primary habitats for *Hydnellum*, particularly in communities dominated by *Quercus*. Therefore, forests dominated by *Pinaceae* (especially *Pinus*) and *Fagaceae* (especially *Quercus*) may represent the preferred habitats for *Hydnellum* in China.

Furthermore, the four new species described in this paper and five important groups (i.e., new discoveries not yet described: *Hydnellum* sp. 1, *Hydnellum* sp. 2, *Hydnellum* sp. 3, *Hydnellum* sp. 4, and *Hydnellum* sp. 5) primarily appear from September to October (Appendix A, Table A1), and most recorded specimens were collected 3–7 days after rainfall, which may indicate their optimal growth stage [51,52]. Moreover, by comparing the collection periods of 44 *Hydnellum* species in China, it was found that they typically appear in autumn, indicating that the distribution of *Hydnellum* species may also be influenced by seasonality [52]. Beyond this temporal window, dry weather and soil water evaporation may inhibit spore germination and mycelial growth, thereby reducing the likelihood of encountering them in ideal habitats and during suitable temporal windows [53,54]. Therefore, detailed field habitat survey records—including host plants, soil and air temperature and humidity, etc.—will help gain an in-depth understanding of the distribution characteristics of species diversity and provide basic data for the assessment and conservation of *Hydnellum* resources in China.

*Hydnellum* is typically rich in diverse bioactive molecules (e.g., phenolic compounds, terpenoids and polysaccharides) that play crucial roles in antiviral, antioxidant, and anti-inflammatory processes, exhibiting high potential for drug development [8,55,56,57]. Phylogenetic analysis reveals that *H. fuscozonatum* and *H. pileospinosum* are closely related to *H. concrescens* and *H. peckii*, respectively, suggesting they may share similar secondary metabolites and pharmacological activities—the latter two have been confirmed to exhibit significant effects in diabetes treatment and anticoagulation [9,56,58]. Additionally, we provided detailed field records for the four new taxa (encompassing habitat environment, tree species, and occurrence time) and systematically summarized the distribution characteristics, host ranges, and reproductive periods of *Hydnellum* species in China. These details are critical for the sustainable collection of fungal materials, as they ensure samples are obtained at the optimal growth stage, thereby supporting subsequent chemical profiling and pharmacological screening. Integrating robust taxonomy (with species identities validated by morphology and DNA barcoding) with ecological context provides a basis for prioritizing these new species as candidate resources for the future isolation of novel bioactive compounds. However, whether the four new species described herein possess pharmacological activities requires further investigation. Future work should involve chemical characterization of their metabolomes and testing the activity of their extracts against disease-related targets (e.g., cancer cell lines, pathogenic microorganisms, inflammatory pathways), which may yield novel lead compounds for diseases with limited current therapies (such as cancer, infections, and inflammation).

**Table 2:** Retrieval table (key) to species of Hydnellum in China.

1. Pileus yellow to orange	2
1. Pileus brown	29
2. Pileus yellow	3
2. Pileus orange	14
3. Spines more or less white	4
3. Spines more or less brown	11
4. Pileus < 30 mm wide	*H. bomiense*
4. Pileus > 30 mm wide	5
5. Pileus ≥ 80 mm wide	6
5. Pileus < 80 mm wide	8
6. Pileus ≥ 100 mm wide	*Hydnellum* sp. 1
6. Pileus 80–100 mm wide	7
7. Habitat in *Abies* and *Pinus* mixed forest	*H. martioflavum*
7. Habitat in *Pinus* and *Quercus* mixed forest	*H. edule*
8. Habitat in August	9
8. Habitat in October	10
9. Habitat in *Picea* forest	*H. caeruleum*
9. Habitat in *Pinus* and *Abies* forest	*H. subscabrosellum*
10. Basidiospores < 5.0 μm long on average	*H. pileospinosum*
10. Basidiospores > 5.0 μm long on average	*Hydnellum* sp. 2
11. Spines > 5.0 μm long on average	*H. subalpinum*
11. Spines < 5.0 μm long on average	12
12. Basidiospores > 5.0 μm long on average	*H. cinnamomea*
12. Basidiospores < 5.0 μm long on average	13
13. Pileus > 40 mm wide	*H. granulosum*
13. Pileus < 40 mm wide	*H. aureoluteum*
14. Pileus light orange to deep orange	15
14. Pileus orange to brown	19
15. Spines dark	16
15. Spines orange	17
16. Spines > 3.0 μm long on average	*H. complicatum*
16. Spines < 3.0 μm long on average	*H. atrospinosum*
17. Spines < 3.0 μm long on average	*H. chrysinum*
17. Spines ≥ 3.0 μm long on average	18
18. Basidiospores ≥ 5.0 μm long on average	*H. earlianum*
18. Basidiospores < 5.0 μm long on average	*H. peckii*
19. Pileus ≥ 70 mm wide	20
19. Pileus < 70 mm wide	25
20. Spines brown	21
20. Spines white	22
21. Habitat in *Picea* forest	*H. ferrugineum*
21. Habitat in *Q. mongolica* forest	*Hydnellum* sp. 4
22. Pileus > 100 mm wide	*H. versipelle*
22. Pileus 70–100 mm wide	23
23. Basidiospores > 5.0 μm long on average	24
23. Basidiospores < 5.0 μm long on average	*H. inflatum*
24. Basidiospores > 7.0 μm long on average	*H. aurantiacum*
24. Basidiospores < 7.0 μm long on average	*H. grosselepidotum*
25. Spines > 3.0 μm long on average	26
25. Spines < 3.0 μm long on average	27
26. Pileus pale orange to dark brown	*H. spongiosipes*
26. Pileus golden yellow to light brown	*H. brunneorubrum*
27. Basidiospores > 5.5 μm long on average	*Hydnellum* sp. 5
27. Basidiospores < 5.5 μm long on average	28
28. Habitat in *Q. mongolica* forest	*Hydnellum* sp. 3
28. Habitat in angiosperm forest	*H. xanthopus*
29. Pileus grayish brown to dark brown	30
29. Pileus light brown to reddish brown	37
30. Pileus grayish brown	31
30. Pileus dark brown	34
31. Basidiospores > 6.0 μm long on average	*H. subailaoensis*
31. Basidiospores < 6.0 μm long on average	32
32. Spines white to brown	*H. fuscozonatum*
32. Spines brown to grayish brown	33
33. Habitat in *Q. acutissima* forest	*H. aureotomentosum*
33. Habitat in mixed forest	*H. chocolatum*
34. Pileus > 60 mm wide	35
34. Pileus < 60 mm wide	36
35. Basidiospores > 5.0 μm long on average	*H. nitidum*
35. Basidiospores < 5.0 μm long on average	*H. sulcatum*
36. Spines brown	*H. melanocarpum*
36. Spines black	*H. radiatum*
37. Pileus light brown	38
37. Pileus reddish brown	44
38. Pileus ≥ 50 mm wide	39
38. Pileus < 50 mm wide	41
39. Basidiospores > 5.0 μm long on average	*H. ailaoense*
39. Basidiospores ≤ 5.0 μm long on average	40
40. Pileus > 100 mm wide	*H. suaveolens*
40. Pileus < 100 mm wide	*H. succulentus*
41. Spines white to brown	42
41. Spines orange brown to reddish brown	43
42. Habitat in *Quercus* forest	*H. fibulatum*
42. Habitat in *Fagaceae* forest	*H. atrorubrum*
43. Basidiospores > 5.0 μm long on average	*H. lidongense*
43. Basidiospores < 5.0 μm long on average	*H. concentricum*
44. Pileus 50–200 mm wide	45
44. Pileus 15–45 mm wide	49
45. Pileus ≥ 90 mm wide	46
45. Pileus < 90 mm wide	47
46. Spines whitish	*H. illudens*
46. Spines grayish brown	*H. fagiscabrosum*
47. Basidiospores > 5.0 μm long on average	48
47. Basidiospores < 5.0 μm long on average	*H. rubidofuscum*
48. Habitat in *Picea* forest	*H. subcaeruleum*
48. Habitat in *Pinus* and *Quercus* mixed forest	*H. crassipileatum*
49. Basidiospores > 5.0 μm long on average	50
49. Basidiospores < 5.0 μm long on average	51
50. Spines white to yellowish white	*H. coactum*
50. Spines layer brown to reddish brown	*H. tardum*
51. Habitat in September	52
51. Habitat in July	*H. squamulosum*
52. Spines white to grayish red	*H. yunnanense*
52. Spines reddish brown	*H. qinghaiense*

## Figures and Tables

**Table 1:** Specimens and sequences used in this study.

Species	Locality	Voucher	ITS	nrLSU	References
*H. ailaoense*	Thailand	HKAS125553 **T**	OP605523	OP602211	[39]
*H. ailaoense*	Thailand	HKAS125554	OP605520	OP602212	[39]
*H. amygdaliolens*	Spain	SC-2011	JN376763	–	[32]
*H. amygdaliolens*	France	GB-0202072	MW144290	MW144290	[32]
*H. atrorubrum*	China	Wei8315	MW579937	–	[31]
*H. atrorubrum*	China	Wei8261 **T**	MW579936	MW579884	[31]
*H. atrospinosum*	China	Yuan6514	MW579940	MW579886	[31]
*H. atrospinosum*	China	Yuan6520 **T**	MW579939	–	[31]
*H. aurantiacum*	Norway	EBendiksen177-07	MK602712	MK602712	[22]
*H. aurantiacum*	Sweden	RGCarlsson08-105	MK602711	MK602711	[22]
*H. aurantiacum*	Czechia	PRM:932984	PV643846	PV682263	[22]
*H. auratile*	Norway	OF242763	MK602715	MK602715	[22]
*H. auratile*	Norway	OF294095	MK602714	MK602714	[22]
***H. aureoluteum***	**China**	**HMJAU-WLB1443a T**	**PX856753**	**PX855234**	**This study**
***H. aureoluteum***	**China**	**HMJAU-WLB1443b**	**PX856754**	**PX855235**	**This study**
***H. aureotomentosum***	**China**	**HMJAU-WLB1431 T**	**PX856755**	**PX855236**	**This study**
***H. aureotomentosum***	**China**	**HMJAU-WLB1437**	**PX856756**	**PX855237**	**This study**
*H. bomiense*	China	Yuan 13759	MW579941	MW579887	[31]
*H. bomiense*	China	Yuan 13767 **T**	MW579942	–	[31]
*H. brunneorubrum*	China	Yuan12997 **T**	MW579944	MW579889	[31]
*H. brunneorubrum*	China	Yuan14339	MW579943	MW579888	[31]
*H. caeruleum*	Norway	EBendiksen584-11	MK602719	MK602719	[22]
*H. caeruleum*	Norway	EBendiksen575-11	MK602718	MK602718	[22]
*H. chocolatum*	China	Cui18545 **T**	ON603657	–	[32]
*H. chocolatum*	China	BJFC 035406	NR185730	–	[32]
*H. chrysinum*	China	SC071	KJ534291	–	[31]
*H. coactum*	China	Wei8094 **T**	MN846278	MN846287	[38]
*H. coactum*	China	Shi181	MN846279	MN846288	[38]
*H. complicatum*	USA	REB-71	KC571711	–	[18]
*H. complicatum*	USA	REB-329	KC571712	–	[18]
*H. concrescens*	USA	SEW 88	AY569025	–	[31]
*H. concrescens*	Mexico	GO-2009-204	KC152116	–	[31]
*H. crassipileatum*	China	Cui17019	ON603659	ON603642	[32]
*H. crassipileatum*	China	Cui17021 **T**	ON603660	ON603641	[32]
*H. cristatum*	USA	REB-169	JN135174	–	[18]
*H. cristatum*	USA	REB-88	KC571718	–	[18]
*H. cyanopodium*	USA	SEW 85	AY569027	–	[31]
*H. dianthifolium*	Cyprus	ML61211HY **T**	KX619419	–	[37]
*H. dianthifolium*	Italy	ML902162HY	KX619420	–	[37]
*H. earlianum*	USA	REB-75	KC571724	–	[18]
*H. earlianum*	USA	REB-375	JN135179	JN135179	[18]
*H. edule*	China	SAAS2870	OK636094	OP407675	[33]
*H. edule*	China	SAAS2920	OK636092	OP407677	[33]
*H. fagiscabrosum*	Sweden	GB-0195805 **T**	MW144294	MW144294	[42]
*H. fagiscabrosum*	Sweden	GB-0195623	MW144295	MW144295	[42]
*H. fennicum*	Norway	OF242833	MK602738	MK602738	[22]
*H. fennicum*	Norway	OF294087	MK602737	MK602737	[22]
*H. ferrugineum*	Sweden	ELarsson197-14	MK602722	MK602722	[22]
*H. ferrugineum*	Sweden	ELarsson 356-16	MK602721	MK602721	[22]
*H. ferrugipes*	USA	REB-176	KC571727	–	[18]
*H. ferrugipes*	USA	REB-58	JN135176	–	[18]
*H. fibulatum*	China	Yuan14656 **T**	MW579958	–	[31]
*H. fibulatum*	China	Yuan14646	MW579957	MW579926	[31]
*H. fuligineoviolaceum*	Sweden	BNylen130918	MK602741	MK602741	[22]
*H. fuligineoviolaceum*	Sweden	LA120818	MK602740	MK602740	[22]
*H. fuscoindicum*	USA	OSC 107844	EU669229	EU669279	[22]
*H. fuscoindicum*	USA	OSC 113622	EU669228	EU669278	[22]
***H. fuscozonatum***	**China**	**HMJAU-TYL3291**	**PX856757**	**PX855238**	**This study**
***H. fuscozonatum***	**China**	**HMJAU-TYL3976 T**	**PX856758**	**PX855239**	**This study**
*H. geogenium*	USA	AFTOL-ID 680	DQ218304	AY631900	[44]
*H. geogenium*	Norway	OF296213	MK602724	MK602724	[22]
*H. glaucopus*	Sweden	UPS-F013955 **T**	MW144330	MW144330	[42]
*H. glaucopus*	Sweden	GB-0195644	MW144319	MW144319	[42]
*H. gracilipes*	Sweden	ELarsson219-11	MK602726	MK602726	[22]
*H. gracilipes*	Sweden	GB-0113779	MK602727	MK602727	[22]
*H. granulosum*	China	Yuan12213b **T**	MW579947	MW579892	[31]
*H. granulosum*	China	Yuan12213a	MW579948	MW579893	[31]
*H. grosselepidotum*	China	Wei8120	MN846274	MN846283	[38]
*H. grosselepidotum*	China	Wei8075	MN846276	MN846285	[38]
*H. illudens*	Sweden	GB-0195819	MW144341	MW144341	[42]
*H. illudens*	Norway	O-F-242769	MW144335	MW144335	[42]
*H. inflatum*	China	Wang80	MW579949	MW579894	[31]
*H. inflatum*	China	Shi506 **T**	MW579950	MW579895	[31]
*H. ioeides*	Sweden	KHjortstam17589	MK602750	MK602750	[22]
*H. ioeides*	Sweden	Nitare110829	MK602751	MK602751	[22]
*H. lepidum*	Sweden	JNitare110829	MK602754	MK602754	[22]
*H. lepidum*	Sweden	RGCarlsson10-065	MK602752	MK602752	[22]
*H. lidongense*	China	We8365 **T**	MN846280	MN846289	[38]
*H. lidongense*	China	Wei8329	MN846281	MN846290	[38]
*H. martioflavum*	Norway	OF242435	MK602754	MK602754	[22]
*H. martioflavum*	Norway	OF242872	MK602752	MK602752	[22]
*H. melanocarpum*	China	Cui18557	ON603662	ON603643	[32]
*H. melanocarpum*	China	Cui18559	ON603663	ON603644	[32]
*H. mirabile*	Sweden	SLund140912	MK602730	MK602730	[22]
*H. mirabile*	Sweden	ELarsson170-14	MK602729	MK602729	[22]
*H. nemorosum*	Norway	O-F-242352	MW144372	MW144372	[42]
*H. nemorosum*	Sweden	GB-0195631	MW144373	MW144373	[42]
*H. parvum*	USA	REB-131	JN135187	–	[18]
*H. parvum*	USA	REB-392	KC571717	–	[18]
*H. peckii*	China	Yuan13708	MW579966	MW579905	[31]
*H. peckii*	China	Yuan13720	MW579967	MW579906	[31]
***H. pileospinosum***	**China**	**HMJAU-TYL3763**	**PX856759**	**PX855240**	**This study**
***H. pileospinosum***	**China**	**HMJAU-TYL4046 T**	**PX856760**	**PX855241**	**This study**
*H. piperatum*	USA	REB-322	JN135173	–	[18]
*H. piperatum*	USA	REB-304	KC571723	–	[18]
*H. radiatum*	China	Cui17130 **T**	ON603664	ON603645	[32]
*H. radiatum*	China	BJFC 030430	NR185733	–	[32]
*H. regium*	USA	SEW-93	AY569031	–	[31]
*H. roseoviolaceum*	Sweden	GB-0195936 **T**	NR185593	MW144374	[42]
*H. roseoviolaceum*	Sweden	GB-0195687	MW144375	MW144375	[42]
*H. rubidofuscum*	China	Yuan14561 **T**	MW579951	MW579896	[31]
*H. rubidofuscum*	China	Yuan14654	MW579953	MW579898	[31]
*H. scabrosellum*	Sweden	GB-0195792	MW144380	MW144380	[42]
*H. scabrosellum*	Sweden	GB-0195689 **T**	MW144379	MW144379	[42]
*H. scabrosum*	Norway	O-F-360777	MK602765	MK602765	[22]
*H. scabrosum*	Norway	O-F-292320	MK602766	MK602766	[22]
*H. scleropodium*	USA	REB-3	JN135186	–	[18]
*H. scleropodium*	USA	REB-352	KC571740	–	[18]
***Hydnellum*** **sp. 1a**	**China**	**HMJAU-TYL566a**	**PX626422**	**PX623214**	**This study**
***Hydnellum*** **sp. 1b**	**China**	**HMJAU-TYL566b**	**PX626423**	**PX623215**	**This study**
***Hydnellum*** **sp. 2a**	**China**	**HMJAU-TYL4119a**	**PX626425**	**PX623218**	**This study**
***Hydnellum*** **sp. 2b**	**China**	**HMJAU-TYL4119b**	**PX626426**	**PX623219**	**This study**
***Hydnellum*** **sp. 3a**	**China**	**HMJAU-TYL285**	**PX626420**	**PX623212**	**This study**
***Hydnellum*** **sp. 3b**	**China**	**HMJAU-TYL503**	**PX626421**	**PX623213**	**This study**
***Hydnellum*** **sp. 4a**	**China**	**HMJAU-TYL200**	**PX626416**	**PX623209**	**This study**
***Hydnellum*** **sp. 4b**	**China**	**HMJAU-TYL204**	**PX626419**	**PX623210**	**This study**
***Hydnellum*** **sp. 4c**	**China**	**HMJAU-TYL608**	**PX626424**	**PX623211**	**This study**
***Hydnellum*** **sp. 5a**	**China**	**HMJAU-TYL200a**	**PX626417**	**PX623216**	**This study**
***Hydnellum*** **sp. 5b**	**China**	**HMJAU-TYL200b**	**PX626418**	**PX623217**	**This study**
*H. spongiosipes*	China	Yuan 14517	MW579968	MW579907	[31]
*H. squamulosum*	China	Yuan 13615 **T**	MW579954	–	[31]
*H. squamulosum*	China	Yuan 13625	MW579956	MW579899	[31]
*H. suaveolens*	Sweden	ELarsson8-14	MK602735	MK602735	[22]
*H. suaveolens*	Norway	SSvantesson877	MK602736	MK602736	[22]
*H. subalpinum*	China	SAAS2778 **T**	OP437919	OP407685	[33]
*H. subalpinum*	China	SAAS2884	OP437920	OP407686	[33]
*H. subsuccosum*	USA	SEW-55	AY569033	–	[31]
*H. subsuccosum*	USA	REB-10	JN135178	–	[18]
*H. sulcatum*	China	Yuan14649 **T**	MW579960	MW579901	[31]
*H. sulcatum*	China	Yuan14660	MW579959	MW579900	[31]
*H. underwoodii*	USA	REB-358	JN135189	–	[18]
*H. underwoodii*	USA	REB-119	KC571782	–	[18]
*H. versipelle*	Sweden	RGCarlsson13-057	MK602771	MK602771	[22]
*H. versipelle*	Sweden	RGCarlsson11-08	MK602772	MK602772	[22]
*H. yunnanense*	China	Yuan14386 **T**	MW579962	MW579903	[31]
*H. yunnanense*	China	Yuan14396	MW579963	MW579904	[31]
*Sarcodon leucopus*	Norway	OF296099	MK602755	MK602755	[31]
*S. leucopus*	Sweden	PHedberg080811	MK602757	MK602757	[22]
*S. scabripes*	Mexico	FCME:23240	EU293829	–	[31]
*S. scabripes*	USA	REB-351	JN135191	–	[18]
*S. squamosus*	Norway	OF177452	MK602768	MK602768	[22]
*S. squamosus*	Norway	OF295554	MK602769	MK602769	[22]

Note: Newly generated sequences in this study are shown in bold; the specimen type is marked by a boldfaced **T**.

## Data Availability

The species registration names and gene accession numbers referenced in this study are publicly available in the following online databases: Fungal Names (https://nmdc.cn/fungalnames/, accessed on 13 January 2026) and NCBI GenBank (https://www.ncbi.nlm.nih.gov/genbank/, accessed on 13 January 2026).

## Appendix A

**Table A1:** Distribution, ecological habits, and key morphological characteristics of *Hydnellum* species in China.

Species	Pileus		Spines		Spores			Habitat	Time	Distribution	References
	Color	Size (mm)	Color	Length (mm)	Size (µm)	av. L × W (µm)	Q				
*H. ailaoense*	light brown to brown	40–75 mm	brown	1.5 mm	5.5–7.0 × 4.5–6.0 μm	6.3 × 5.2 μm	Q = 1.09–1.33	*Fagaceae* and *Pinaceae* mixed forests	August	Yunnan, China	[39]
*H. atrorubrum*	light brown to dark ruby	48 mm	white to dark brown	3.5 mm	4.5–6.0 × 3.9–5.1 μm	5.0 × 4.4 μm	Q = 1.14–1.21	*Fagaceae* forest	July	Yunnan, China	[31]
*H. atrospinosum*	light orange to yellowish brown	75 mm	dark violet	2.5 mm	4.1–5.1 × 3.1–3.9 μm	4.6 × 3.2 μm	Q = 1.34–1.44	*Picea* forest	September	Qinghai, China	[31]
*H. aurantiacum*	orange to deep orange	100 mm	white	4.0 mm	5.5–8.0 × 5.5–6.5 μm	8.2 × 6.2 μm		hardwood forests	July to November	China, USA	[20]
*H. aureoluteum*	golden yellow	16.5–30.5 mm	grayish orange to light brown	0.5–1.5 mm	4.0–5.0 × 4.0–4.5 μm	4.57 × 4.03 μm	Q = 1.00–1.25	*Q. acutissima* forest	July	Sichuan, China	This study
*H. aureotomentosum*	gray to brown	19.5–75.5 mm	brown	2.0–4.5 mm	5.5–6.0 × 5.0–5.5 μm	5.91 × 5.04 μm	Q = 1.00–1.20	*Q. acutissima* forest	July	Sichuan, China	This study
*H. bomiense*	yellow brown to dark brown	26 mm	white to brown	1.1 mm	4.1–5.1 × 3.3–4.5 μm	4.7 × 4.0 μm	Q = 1.18–1.21	*Fagaceae* forest	July to September	Xizang, China	[31]
*H. brunneorubrum*	brownish orange to brownish red	40 mm	golden yellow to light brown	4.0 mm	4.1–5.1 × 3.2–4.6 μm	4.9 × 3.9 μm	Q = 1.23–1.26	*Fagaceae* forest	August	Liaoning, China	[31]
*H. caeruleum*	pastel yellow to dark blonde	–	orange white to dark brown	6.0 mm	5.0–6.0 × 4.1–4.9 μm	–	–	*Picea* forest	August	China, North Europe, and temperate Asia	[31]
*H. chocolatum*	chocolate to fuscous	74 mm	brown to grayish brown	5.0 mm	5.0–6.0 × 4.0–5.0 μm	5.2 × 4.7 μm	Q = 1.00–1.25	mixed forest	September	Sichuan, China	[32]
*H. chrysinum*	light orange to mars orange	70 mm	light orange to salmon orange	2.0 mm	4.0–6.0 × 3.2–5.0 μm	–	–	–	–	–	[34]
*H. cinnamomea*	yellowish brown to brown	62 mm	brown to grayish brown	3.0 mm	4.5–6.0 × 4.0–5.0 μm	5.2 × 4.5 μm	Q = 1.14–1.16	*Quercus* forest	September	Sichuan, China	[34]
*H. coactum*	reddish brown to dark brown	35 mm	white to yellowish white	2.1 mm	5.7–7.0 × 4.7–5.9 μm	6.2 × 5.3 μm	Q = 1.17–1.18	*Fagaceae* forest	July/August	Yunnan, China	[38]
*H. complicatum*	white, orange white to light orange	60 mm	carrot red to brusing black	4.0 mm	4.0–5.0 × 3.0–5.0 μm	4.0 × 3.0 μm	–	–	June to September	China, USA	[34]
*H. concentricum*	light brown to grayish brown	32 mm	reddish brown	3.0 mm	4.0–5.0 × 3.5–5.0 μm	4.6 × 3.9 μm	Q = 1.04–1.37	*Pinus* and *Quercus* mixed forest	September	Yunnan, China	[32]
*H. crassipileatum*	reddish brown to grayish brown	55 mm	grayish brown	5.0 mm	4.0–6.0 × 4.0–5.5 μm	5.6 × 4.5 μm	Q = 1.00–1.38	*Pinus* and *Quercus* mixed forest	September	Yunnan, China	[32]
*H. earlianum*	light orange to deep orange	70–90 mm	cream to brownish orange	4.0 mm	5.0–6.0 × 4.0–5.0 μm	4.7 × 5.0 μm	–	riparian broadleaf mixed forest	June/August	China, USA	[34]
*H. edule*	brownish yellow to reddish brown	50–90 mm	whitish	8.0 mm	5.5–7.0 × 4.5–5.5 μm	–	–	*Pinus* and *Quercus* mixed forest	July/August	Sichuan, China	[33]
*H. fagiscabrosum*	red brown to black brown	50–140 mm	grayish brown	–	4.5–6.3 × 3.8–5.3 μm	5.4 × 4.7 μm	Q = 0.90–1.50	*Fagaceae* forest	July to Octomber	China, Norway, Sweden, the UK, Italy and USA	[34]
*H. ferrugineum*	pale orange to brown	80 mm	brownish gray to burnt umber	6.0 mm	5.0–6.0 × 5.0–6.0 μm	6.0 × 5.0 μm	–	*Picea* forest	August	China, USA	[34]
*H. fibulatum*	light brown to dark brown	45 mm	pinkish white to brown	1.5 mm	4.4–5.8 × 4.1–4.9 μm	5.2 × 4.3 μm	Q = 1.12–1.21	*Quercus* forest	August	Liaoning, China	[31]
*H. fuscozonatum*	gray to brownsh gray	12.5–65.4 mm	white to brown	0.5–2.5 mm	5.5–6.0 × 5.0–5.5 μm	5.94 × 5.02 μm	Q = 1.09–1.20	*Q. glauca* forest	September/October	Anhui, China	This study
*H. granulosum*	light yellow to grayish brown	50 mm	grayish orange to dark brown	2.0 mm	4.1–5.1 × 3.4–4.7 μm	4.6 × 4.1 μm	Q = 1.12–1.13	*Acer* and *Cryptomeria* mixed forest	August	Sichuan, China	[31]
*H. grosselepidotum*	pale orange to dark ruby	75 mm	white to pale yellow	1.4 mm	5.1–6.4 × 4.1–5.9 μm	5.5 × 4.9 μm	Q = 1.13–1.19	*Fagaceae* forest	July/August	Yunnan, China	[38]
*H. illudens*	ochraceous to fulvous brown	60–100 mm	whitish	5.0 mm	5.0–6.5 × 4.5–6.0 μm	–	–	*Quercus* forest	August	Sichuan, China; France	[33]
*H. inflatum*	grayish orange to brown	75 mm	white to golden brown	4.0 mm	4.2–5.0 × 3.8–4.3 μm	4.8 × 4.0 μm	Q = 1.18–1.20	*Fagaceae* forest	October	Yunnan, China	[31]
*H. lidongense*	light brown to brown	35 mm	grayish orange to brown	1.0 mm	4.1–6.0 × 4.0–5.0 μm	5.5 × 4.9 μm	Q = 1.15–1.20	*Fagaceae* forest	July	Yunnan, China	[38]
*H. martioflavum*	yellow brown to light brown	90 mm	gray whitish to gray	5.0 mm	5.0–6.5 × 4.0–5.5 μm	–	–	*Abies* and *Pinus* mixed forest	September	Sichuan, China; Canada	[33,38]
*H. melanocarpum*	brown to black	48 mm	brown	4.0 mm	4.5–5.5 × 3.8–5.1 μm	5.0 × 4.6 μm	Q = 1.00–1.25	mixed forest	September	Sichuan, China	[32]
*H. nitidum*	grayish brown to dark brown	110 mm	reddish brown to grayish brown	4.0 mm	4.8–6.0 × 4.0–5.0 μm	5.2 × 4.7 μm	Q = 1.08–1.20	mixed forest	September	Sichuan, China	[34]
*H. peckii*	white to light orange	–	brownish orange	3.0 mm	4.2–5.1 × 3.9–4.4 μm	–	–	*Pinus* mixed forest	July	China, North Europe, Iran, and Korea	[31]
*H. pileospinosum*	grayish yellow to yellowish brown	26.2–60.3 mm	white	1.0–6.7 mm	4.0–5.0 × 4.0–4.5 μm	4.41 × 4.02 μm	Q = 1.00–1.13	*Q. Glauca* forest	October	Anhui, China	This study
*H. qinghaiense*	reddish brown	40 mm	grayish blue to brown	4.0 mm	4.0–5.0 × 3.0–4.0 μm	4.4 × 3.8 μm	Q = 1.1–1.24	*Picea crassifolia* forest	September	Qinghai, China	[34]
*H. radiatum*	dark brown to black	29 mm	black	3.0 mm	4.0–5.0 × 3.0–4.5 μm	4.5 × 3.8 μm	Q = 1.00–1.35	–	September	Yunnan, China	[32]
*H. rubidofuscum*	reddish brown	70 mm	grayish brown to reddish brown	3.0 mm	4.1–5.0 × 3.9–4.6 μm	4.6 × 4.1 μm	Q = 1.11–1.12	*Quercus* forest	August	Liaoning, China	[31]
*Hydnellum* sp. 1	brownish yellow to dark brown	62.5–140.5 mm	white to yellowish white	4.8–8.5 mm	6.0–6.5 × 5.0–5.5 μm	6.2 × 5.1 μm	Q = 1.12–1.16	*P. koraiensis* forest	September	Jilin, China	This study
*Hydnellum* sp. 2	brownish yellow	63.6–80.5 mm	white to yellowish white	2.0–6.2 mm	5.0–6.0 × 5.0–6.0 μm	5.3 × 5.2 μm	Q = 1.00–1.10	*Q. serrata* forest	October	Anhui, China	This study
*Hydnellum* sp. 3	brownish orange to dark brown	17.6–42.3 mm	light orange to light brown	0.5–2.5 mm	5.0–5.5 × 4.0–5.0 μm	5.0 × 4.7 μm	Q = 1.00–1.25	*Q. mongolica* forest	August/September	Jilin, China	This study
*Hydnellum* sp. 4	brownish orange to dark brown	32.5–100.6 mm	light orange to light brown	0.5–2.5 mm	6.0–7.0 × 6.0–6.5 μm	6.7 × 6.2 μm	Q = 1.00–1.16	*Q. mongolica* forest	September	Jilin, China	This study
*Hydnellum* sp. 5	orange to brownish yellow	8–15 mm	brownish yellow	0.5–1.0 mm	5.5–6.0 × 5.0–6.0 μm	5.9 × 5.1 μm	Q = 1.00–1.25	*Q. variabilis* forest	July	Jilin, China	This study
*H. spongiosipes*	pale orange to dark brown	–	pale orange to dark brown	6.0 mm	5.1–6.1 × 4.5–5.3 μm	–	–	*Quercus* forest	August	China, USA, and Europe	[31]
*H. squamulosum*	pastel red to dark magenta	35 mm	pale red to reddish brown	2.0 mm	4.1–5.0 × 3.3–4.1 μm	4.4 × 3.8 μm	Q = 1.14–1.16	*Picea* mixed forest	July	Xizang, China	[31]
*H. suaveolens*	light brown to brownish	160 mm	light brown to dark brown	5.0 mm	4.0–6.0 × 3.0–5.0 μm	5.0 × 4.0 μm	–	*Picea* forest	August	China, USA	[34]
*H. subailaoensis*	brown to grayish brown	62 mm	light vinaceous gray	3.0 mm	6.0–7.5 × 5.0–6.5 μm	6.4 × 5.6 μm	Q = 1.12	–	October	Yunnan, China	[34]
*H. subalpinum*	brownish yellow to brown	50–200 mm	concolorous	8.0 mm	3.5–4.5 × 4.0–5.5 μm	–	–	*Abies* forest	September	Sichuan, China	[33]
*H. subcaeruleum*	reddish brown to dark brown	50 mm	olivaceous buff to clay buff	4.0 mm	4.5–6.0 × 4.0–5.0 μm	5.2 × 4.5 μm	Q = 1.13–1.19	*Picea* forest	September	Gansu, China	[34]
*H. subscabrosellum*	brownish yellow to reddish brown	30–70 mm	whitish to yellowish	5.0 mm	5.0–7.0 × 4.5–6.0 μm	–	–	*Pinus* and *Abies* mixed forest	August	Sichuan, China	[33]
*H. succulentus*	light brown to brown	50 mm	pale reddish brown	4.0 mm	4.0–5.0 × 4.0–5.5 μm	4.9 × 4.4 μm	Q = 1.10–1.19	angiosperm forest	June/August	Yunnan, China	[34]
*H. sulcatum*	dark brown	65 mm	brown	1.5 mm	4.1–5.8 × 4.0–4.6 μm	4.8 × 4.3 μm	Q = 1.14–1.19	*Quercus* forest	August	Liaoning, China	[31]
*H. tarda*	dark reddish brown	35 mm	layer brown to reddish brown	3.0 mm	4.5–6.0 × 4.0–5.0 μm	5.2 × 4.4 μm	Q = 1.17–1.20	*Fagaceae* forest	June	Yunnan, China	[34]
*H. versipelle*	brownish orange to reddish brown	200 mm	whitish	9.0 mm	4.0–5.5 × 3.0–4.0 μm	–	–	*Abies* forest	September	Sichuan, China; Europe	[33,38]
*H. xanthopus*	dawn to orange brown	24 mm	clay buff	2.0 mm	3.5–4.8 × 4.0–5.5 μm	4.9 × 4.3 μm	Q = 1.14	angiosperm forest	August	Sichuan, China	[34]
*H. yunnanense*	grayish red to dark brown	21 mm	white to grayish red	1.5 mm	4.2–5.1 × 3.5–4.5 μm	4.7 × 4.0 μm	Q = 1.17–1.18	*Pinus* forest	September	Yunnan, China	[31]
